# Excretion patterns of *Schistosoma mansoni* antigens CCA and CAA by adult male and female worms, using a mouse model and *ex vivo* parasite cultures

**DOI:** 10.1017/S0031182021001839

**Published:** 2022-03

**Authors:** Miriam Casacuberta-Partal, Lisette van Lieshout, Angela van Diepen, Jeroen C. Sijtsma, Arifa Ozir-Fazalalikhan, Jan Pieter R. Koopman, Claudia J. de Dood, Paul L.A.M. Corstjens, Govert J. van Dam, Cornelis H. Hokke, Meta Roestenberg

**Affiliations:** 1 Department of Parasitology, Leiden University Medical Centre, P4-Q, PO Box 9600, 2333 ZA Leiden, The Netherlands; 2 Department of Cell and Chemical Biology, Leiden University Medical Center, 2333 ZA Leiden, The Netherlands; 3 Department of Infectious Diseases, Leiden University Medical Center, 2333 ZA Leiden, The Netherlands.

**Keywords:** Animal infection model, circulating anodic antigen (CAA), circulating cathodic antigen (CCA), mice, monosexual infection, *S. mansoni*, serum, urine, worm culture

## Abstract

Assays which enable the detection of schistosome gut-associated circulating anodic (CAA) and cathodic (CCA) antigen in serum or urine are increasingly used as a diagnostic tool for schistosome infection. However, little is known about the production and clearance of these circulating antigens in relation to the sex and reproductive maturity of the parasite. Here we describe CAA and CCA excretion patterns by exploring a mouse model after exposure to 36 male-only, female-only and mixed (male/female) *Schistosoma mansoni* cercariae. We found that serum and urine CAA levels, analysed at 3 weeks intervals, peaked at 6 weeks post-infection. Worms recovered after perfusion at 14 weeks were cultured *ex vivo*. Male parasites excreted more circulating antigens than females, in the mouse model as well as *ex vivo*. In mixed infections (supporting egg production), serum CAA levels correlated to the number of recovered worms, whereas faecal egg counts or *Schistosoma* DNA in stool did not. No viable eggs and no inflammation were seen in the livers from mice infected with female worms only. *Ex vivo*, CAA levels were higher than CCA levels. Our study confirms that CAA levels reflect worm burden and allows detection of low-level single-sex infections.

## Graphical abstract

**Figure:**
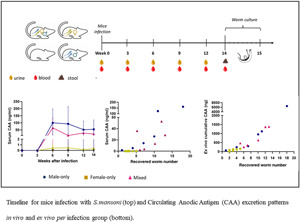

## Introduction

*Schistosoma mansoni* is one of the main schistosome species infecting human beings (Colley *et al*., 2014). Snail-derived cercariae penetrate the human skin and migrate into the vascular system, where mature male and female worms reside in the mesenteric and hepatic veins, where they mate and produce ~300 eggs per day per pair provoking inflammatory responses, which can lead to periportal fibrosis and portal hypertension (Manzella *et al*., 2008; McManus *et al*., 2018).

Due to their intravascular localization, worms cannot be directly quantified in infected humans, therefore the diagnosis of *S. mansoni* infections is traditionally based on the detection of schistosome eggs in the stool. Due to the fluctuation of egg counts, microscopy of a single stool sample is unreliable for quantification of infection intensity. In addition, low burden infections are often missed (Berhe *et al*., 2004). An alternative diagnostic method for schistosome infections is the detection of circulating antigens. Circulating anodic (CAA) and cathodic (CCA) antigen are produced in the gut of the parasite, from where they are regurgitated into the blood circulation of the host and excreted *via* the kidneys into the urine (Deelder *et al*., 1980). The full details of the dynamics of these two heavily glycosylated antigens are not clear, i.e. the rate and possible irregularity of antigen production, the genes involved, its metabolism and biodistribution in the host, and clearance from the blood circulation. Nevertheless, several animal studies have shown a good correlation between worm loads and CAA levels (Barsoum *et al*., 1990; Agnew *et al*., 1995; Alan Wilson *et al*., 2006). Also in humans, different population studies have shown a significant correlation between antigen levels and egg excretion (Kremsner *et al*., 1993; Van Lieshout *et al*., 1995). Compared to the highly fluctuating excretion of eggs in the stool, the levels of CAA and CCA are generally considered a better and more stable measure of the effect of chemotherapy when followed over time (Langenberg *et al*., 2020; Hoekstra *et al*., 2021). However, to aid interpretation of circulating antigen levels as a direct reflection of worm load, the excretion of CAA and CCA needs to be further studied both in single and in paired infections, in particular in relation to their stage of development.

The adult worms exhibit sexual dimorphism, the male holds the female within the mesenteric venules of the human host. It has been previously reported that reproductive development in female worms is dependent on the presence of the male (Lu *et al*., 2017). As a consequence, virgin females have underdeveloped vitellaria and ovaries, which may impact tegumental uptake characteristics and consequently digestive activity. Moreover, because of the incomplete sexual development, unpaired females have been described to be unable to lay eggs (Kunz, 2001; Pearce and Huang, 2015). Both CAA and CCA are excreted by juveniles as well as adult worms. It remains unknown whether the reproductive maturity of the schistosomes affects antigen excretion. Particularly in low-level infections, where male or female worms may not meet and pair, the potential inability of these unpaired worms to excrete antigens in similar amounts as paired worms may be relevant to guide therapy.

Recently, a single-sex male *S. mansoni* infection model has been developed in humans to facilitate the expedited testing of novel schistosome vaccines (Langenberg *et al*., 2020). Given the selective expression of an important vaccine target antigen Smp80 by female worms (Siddiqui and Siddiqui, 2017; Zhang *et al*., 2018), the possibility of a controlled human female schistosome infection model is currently being explored. An important consideration when developing these models is the potential difference in the relative capacity of male and female schistosomes to produce CCA and CAA as detection of these antigens in blood or urine are the only means of detecting single-sex infections that lack egg production so far.

To address this consideration the current study aimed to investigate CAA and CCA excretion by single-sex unmated and paired male/female worms to gain insights in the antigen excretion patterns, in particular in relation to the sex and development stage of the parasite. Additionally, we aimed to confirm the absence of mature eggs and liver inflammation in a single-sex infection.

## Materials and methods

### Animal infection

Mice (C57BL/6JOlaHsd) were kept in individually ventilated cages in the animal facility of the Leiden University Medical Center (Leiden, The Netherlands). All mice were housed in a temperature-controlled room with a 12-hour light-dark cycle with *ad libitum* access to food and water and all were used for experiments at 7 weeks of age.

**Fig. 1.**
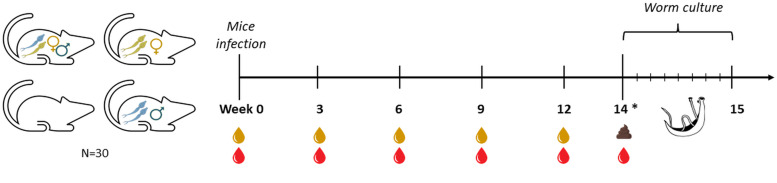
Schematic representation of the experimental timeline. At week 0 mice were infected with 36 cercariae of *S. mansoni*. At weeks 0, 3, 6, 9, and 12 serum (red, 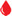) and urine samples (yellow, 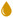) were collected. At week 14 (*) animals (*n* = 22) were sacrificed, serum and stool (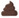) samples were collected, while worms were recovered and cultured *per* animal in a single well over a period of 8 days to assess antigen excretion patterns.

The study included 30 mice in total, of which six served as uninfected controls (C). The other 24 were percutaneously infected with 36 *S. mansoni* cercariae shed from *Biomphalaria glabrata* snails. The sex of cercariae was determined by a purpose-made multiplex real-time polymerase chain reaction (PCR) targeting *Schistosoma*-specific ITS2 sequences, as well as the *S. mansoni* W1 repeats as previously reported (Janse *et al*., 2018). Eight mice were infected with male-only (M) cercariae, eight with female-only (F) cercariae and eight with an assumed 50%–50% mix of males and females (M + F) cercariae. Urine and serum samples from all mice were collected for antigen detection measurements at baseline, week 3, 6, 9 and 12 after infection (Fig. 1). Six out of eight infected mice per group and four out of six control mice were used for perfusion at week 14. At perfusion, additional serum samples were collected for antigen detection before collecting the worms from the hepatic veins for *ex vivo* culture (Mann *et al*., 2010). After perfusion, the livers and intestines were taken for egg isolation and histology, and stool samples were obtained for microscopy and detection of *Schistosoma*-specific DNA by PCR after 12 or 14 weeks. Further details are explained below.

### Egg isolation and histology from liver and intestines

Collected livers were weighed individually (Supplementary, Table 1) and cut into smaller pieces for (i) egg hatching assays, (ii) egg counting, and (iii) histology. For the hatching assay (i), each sample was crushed, filtered and washed with bottled water (Bar-le-Duc Mineral water) to obtain a homogenate of 4 ml. Following exposure to artificial light for 3 hours, the number of miracidia was counted. Tissue digestion (ii) was performed by mincing each sample with 5% potassium hydroxide (KOH) followed by an overnight incubation 37°C. Following centrifugation (400 × g, 5 minutes), the supernatant was discarded, after which the pellet was resuspended in PBS. Centrifugation and resuspension steps were repeated until the supernatant was clear. Lastly, the full pellet was examined for the presence of eggs.

Intestines were collected and weighed (Supplementary, Table 1). From each mouse, a piece of 2 cm long was used for histological examination, while the rest of the intestine was digested and examined for the presence of eggs using the same procedure as for the liver except using a sieve of 80 *μ*m. Histology sections (iii) from liver and intestine tissues were fixed in 4% paraformaldehyde and embedded in paraffin. The tissue samples were sectioned at 4 *μ*m with a Histocore multicut microtome (149MULTI0C1, Leica) and transferred to glass slides and allowed to dry for 24 h. Sections were stained with hematoxylin and eosin (Cardiff *et al*., 2014). Pictures were taken by using 10 × magnification using a Pannoramic 250 Flash II slidescanner (3DHISTECH).

### Egg counts in stool

Stool samples were examined by microscopy by preparing faecal slides within a few hours following perfusion. One Kato-Katz (KK) thick smear slide was prepared per stool sample, using a 25 mg template (Polderman *et al*., 1985). Total egg counts were recorded and then multiplied by a factor of 40 to obtain the number of eggs per gram of faeces (EPG).

### *Schistosoma* PCR

A single stool sample collected at perfusion point or at week 12 were included in this analysis (Fig. 1). To detect the presence of *Schistosoma* DNA in stool, a real-time PCR analysis targeting the internal transcribed spacer (ITS-2) gene was performed. Sample handling, DNA extraction and PCR analysis were performed as previously described (Meurs *et al*., 2015).

### Culture of adult worms

Adult worms were washed and cultured in Dulbecco's modified Eagle's medium (DMEM) (Thermo Fisher Scientific, the Netherlands), supplemented with 10% foetal bovine serum and 2% antibiotic/antimycotic solution (Merck KGaA, Darmstadt, Germany). Worms were dispensed in 6-well plates (Thermo Fisher Scientific, the Netherlands) in 1 mL DMEM medium using one well per perfused animal and were cultured for 8 days at 37°C and 5% CO_2_. Every 24 h, the culture medium was collected, stored for further circulating antigen detection and refreshed with new medium. Every day each individual well was microscopically examined for worm survival assessment and egg deposition. Dead worms were not removed from the cultures.

### Circulating antigen tests

Worm culture media, mouse serum and urine samples were stored at −20°C until testing for circulating antigen levels. Due to limited sample volumes, serum and urine samples could be tested for CAA only, while worm culture medium was tested for both CAA and CCA. The applied antigen test was the lateral flow (LF) based test with luminescent up-converting reporter particles (UCP) allowing quantitative detection of both antigens (Corstjens *et al*., 2014, 2020). In brief, for the UCP-LF CAA and CCA assay 50 *μ*L aliquots (diluted serum and urine samples) were mixed with 50 *μ*L of 4% (w/v) trichloro-acetic acid (TCA) and centrifuged in order to remove interfering proteins and to dissociate potential immune complexes as indicated in Corstjens *et al*. (2020). Extracted samples were incubated with the UCP-antibody conjugate solution specific for either CAA or CCA. The mixture was then run on appropriate LF strips. Upon drying, LF strips were scanned for UCP reporter signals with a for UCP detection adapted Packard FluoroCount strip reader. A standard dilution curve of TCA soluble fraction of adult worm antigen (AWA-TCA) with known CAA/CCA concentrations was used as a reference standard (Corstjens *et al*., 2014). Results from both UCP-LF assays were expressed as ng/ml CAA or CCA.

### Statistical analyses

The outcome was presented as the mean and standard deviation for multiple samples. All statistical analyses were performed using SPSS version 26 (IBM Nederland BV). For comparison of groups, unpaired t-tests, or for non-normal distributions, non-parametric tests Mann–Whitney U tests were performed. For association measurements between two variables, Spearman rank-order correlation coefficient was used. Differences between groups were considered statistically significant at *P* < 0.05.

## Results

**Fig. 2.**
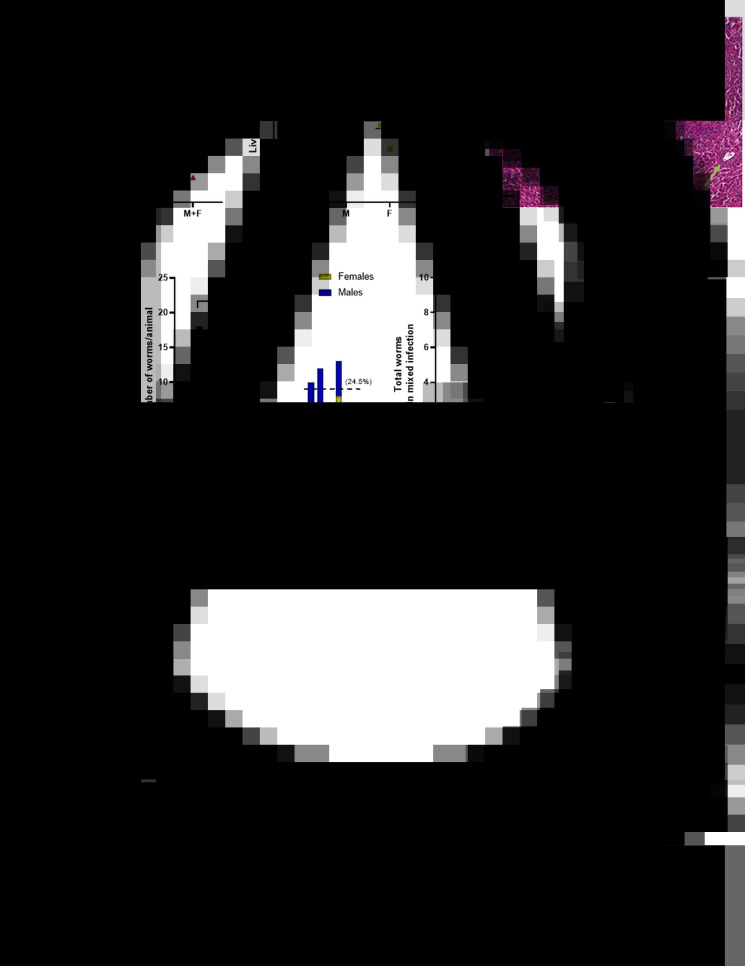
Weight (gr) of mice (*n* = 30) (A) and livers (*n* = 22) (B) according to infection group (Control, male-only, female-only and mixed infection) 14 weeks after infection with 36 cercariae of *S. mansoni*. The solid line represents the mean of each group. Section of a liver at perfusion time (scale bar 25 *μ*m) following H-E staining from a mixed infected mouse. Arrows indicate eggs trapped in a granuloma (C). Number of adult worms recovered from each infected animal *per* group (male-only, female-only and mixed infection). The dashed line represents the mean worm *per* group and the average (%) of the worm recovery rate *per* group (D). Association of a total number of females (yellow triangle) and males (blue triangle) recovered worms (from mixed infected mice only) with the number of eggs *per* gram of digested liver tissue (E). *P < 0.01, **P = 0.02.

Following infection with 36 male (M), female (F) or mixed (M + F) *S. mansoni* cercariae, the total body weight of each individual mouse was monitored. At week 14 after infection, no significant difference was found between the M, F or M + F group (Fig. 2A). However, at 14 weeks after infection, livers of mice infected with M + F parasites had a significantly higher weight (mean 1.9 gr; s.d. 0.1) than livers of mice from M (1.25 gr; s.d. 0.05; *P* = 0.002), F (1.26 gr; s.d. 0.08; *P* = 0.004) and non-infected control (C) animals (1.08 gr; s.d. 0.04; *P* = 0.009) (Fig. 2B).

Eggs were counted in a digested tissue sample of the collected livers and intestines, showing a mean of 9,448 eggs per gram of liver tissue (|s.d. 2380) and a mean of 2041 eggs per gram of intestinal tissue (s.d. 982) in M + F mice (Supplementary, Table 1). No eggs were recovered following digestion of liver or intestinal tissue of the M mice, while examination of the tissues in F mice revealed two eggs in one single liver sample from one mouse. These eggs, although normal in size and showing the characteristic lateral spine, presented as unviable with a darkened and granular content without any signs of a miracidium. An egg hatching assay was performed on a liver lobe and active miracidiae were observed for the M + F group while no miracidiae were observed in any of the other infection groups.

Histology sections of all 22 livers and intestines were performed to assess inflammation caused by egg deposition. Livers (Fig. 2C) and intestines from M + F mice showed granuloma formations around eggs trapped in the tissue, while no eggs were seen in the histology sections of the organs collected from the other animals.

Stool examination by KK at perfusion revealed *S. mansoni* eggs in only two mice, both from M + F group (mean 40 EPG), while no eggs were found in any of the other examined stool samples. However, using more sensitive molecular detection methods, *Schistosoma* specific DNA was detected in the stool of all 8 M + F mice (mean Ct-value 21.6, range 20.3–23.5), while the remaining 22 stool samples were PCR negative. No correlation was seen between Ct-value and egg numbers in faeces or in the organs, or a number of recovered worms. The two microscopy positive stool samples showed a Ct-value of 20.9 and 23.1.

At perfusion, the mean number of worms recovered from M + F infected mice (8.8, s.d.: 3.3) was significantly higher (*P* = 0.02) than the worms recovered from F group (3.0, s.d.: 1.2) but not compared to the M group (8.5, s.d.: 6) (Fig. 2D, dashed lines). Based on an infection dose of 36 cercariae, the highest worm recovery was in the M + F and the M group with a mean of 24.5% (s.d.: 9) and 23.6% (s.d.: 16), respectively, followed by 8.30% (s.d.: 3) at the F group (Fig. 2D). Notably, of the recovered worms in the M + F group on average more were female worms (56.3%) than male worms (43.6%). The number of recovered worms in the M + F group, either in total or females only, was not correlated to the total number of eggs per gram of liver (Fig. 2E) nor of intestine tissues in the M + F group. At perfusion, all worms (M, F, M + F) were found to be motile. Worms from mixed infections were not paired once cultured *ex vivo*.

**Fig. 3.**
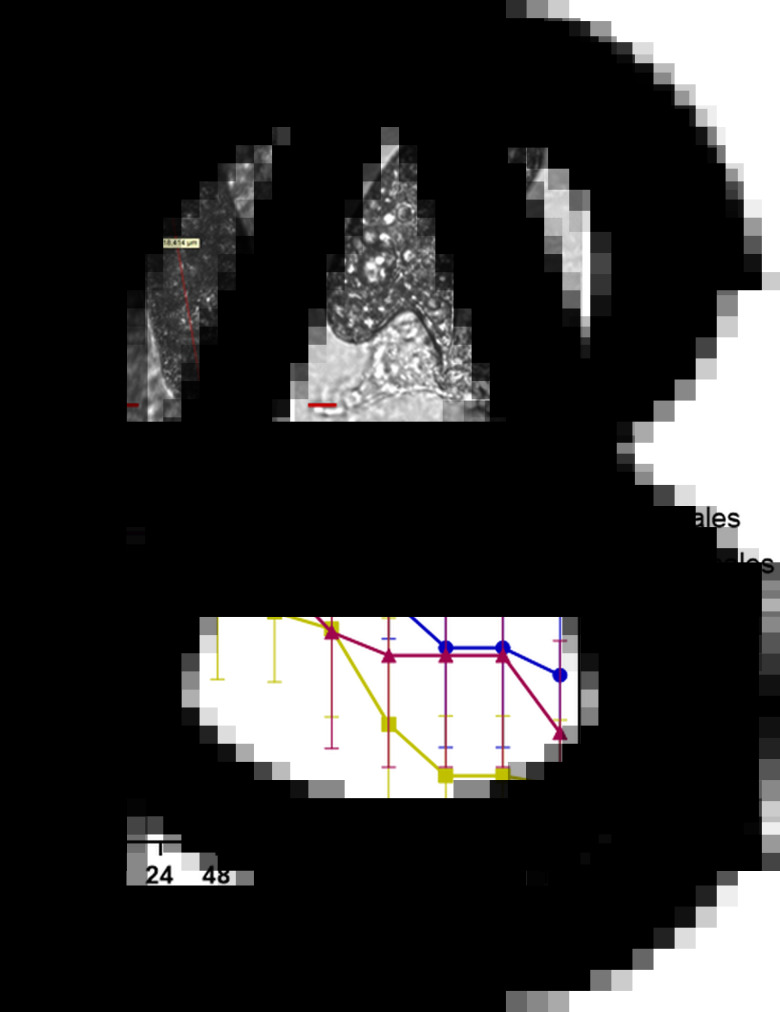
White field microscopy images of a non-viable egg found during *ex vivo* culture of worms obtained from one female-infected mouse. Length of the egg shell 118.45 *μ*m (scale bar 10 *μ*m) (A). Lateral spine of the egg shell (B). Worm survival over the 8 days of *ex vivo* culturing in DMEM (37 ℃). M: blue, circle; F: yellow, square; M + F: pink, triangle (C).

During *ex vivo* culture of the M + F worms, all wells showed high egg deposition in the culture media with the eggs showing a normal morphology. In the F culture, only one egg was found, which was normal in size and with the characteristic lateral spine, but appeared non-viable, darkened and granular and showed no miracidium (Fig. 3A and B).

Survival assessment of worms during *ex vivo* culture showed that in the M group a total of 94% of the worms survived up to 72 h, compared to a 74% in the F group and 87% in the M + F group. After this time, worm viability dropped to 55%, 20% and 35% for the M, F and M + F groups at 8 days respectively. A large variation in worm survival was seen between the individual culture wells (Fig. 3C).

### Serum and urine CAA excretion

**Fig. 4.**
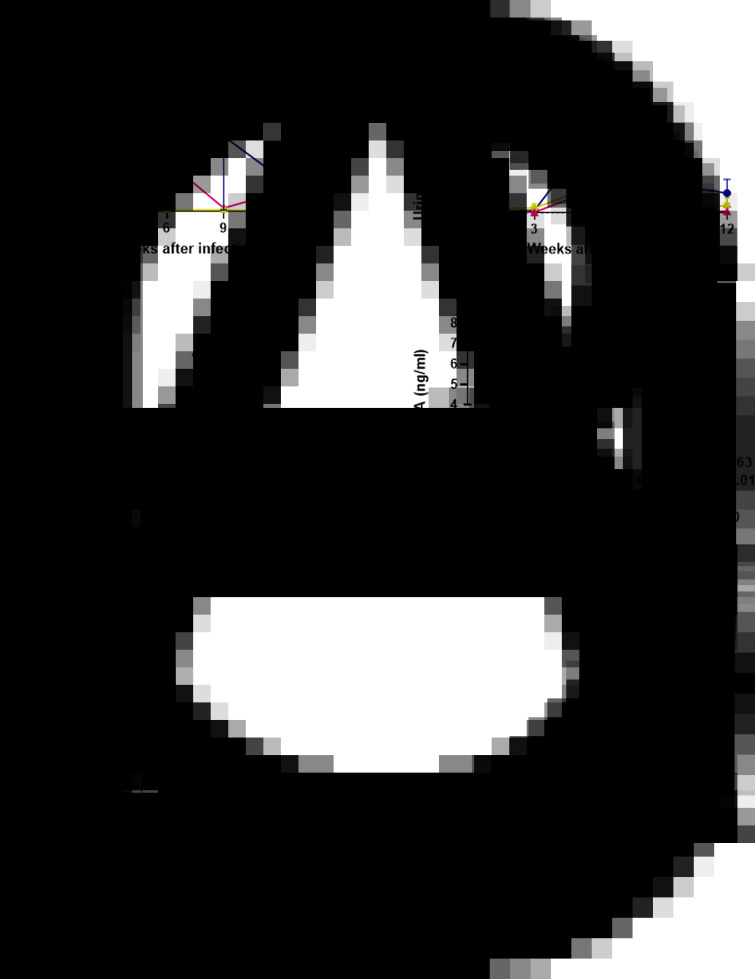
Mean concentration (ng/mL) of CAA *per* recovered worm collected at week 14 in serum (A) and week 12 in urine (B) for the different groups. Association between CAA levels detected in serum (C) and urine (D) at week 12 with total number of worms recovered at perfusion. M: blue, circle; F: yellow, square; M + F: pink, triangle; Control: dashed line. Note the axis have different scales.

Mouse serum and urine CAA levels were determined by UCP-LF assay every three weeks after infection (Fig. 4; Supplementary Fig. 1). Mean CAA levels corrected *per* number of recovered worms increased up to week 6 after infection (Fig. 4A and B), thereafter CAA levels tended to decline specially for the M + F infected mice. In serum, at week 6, the M infection group showed the highest level of CAA *per* worm (mean 11.4 ng/mL s.d.: 7.5) which was roughly 1.6 fold higher than M + F infected mice (mean 7.08 ng/mL s.d.: 8.8) and roughly 80 fold higher than F infected mice (mean 0.14 ng/mL s.d.: 0.14) (Fig. 4A).

Antigen concentration in urine showed a similar pattern as in serum, although overall concentrations *per* worm were lower for M and M + F groups (week 6, M mean 1.26 ng/mL s.d.: 0.7; M + F mean 0.36 s.d.: 0.4) but slightly higher for the F group (week 6, mean 0.42 ng/mL s.d.: 0.4) (Fig. 4B), compared to the respective serum concentrations. At week 6, the M infection group showed a higher level of CAA in urine compared to the F group (*P* = 0.004). Overall, M infected mice showed the highest CAA concentration per worm recovered, both in serum and urine.

Serum CAA concentrations measured at week 14 also correlated with the total number of worms recovered (*r* = 0.82, *P* < 0.05, data not shown). For a better comparison between serum and urine, we used the 12-week timepoint since urine was not available at week 14. CAA concentrations were analysed at week 12 which revealed a better correlation between serum CAA and the total number of worms recovered at perfusion (*r* = 0.83; *P* < 0.001) than in urine CAA (*r* = 0.63; *P* = 0.01) (Fig. 4C and D).

### Worm culture CAA and CCA excretion

**Fig. 5.**
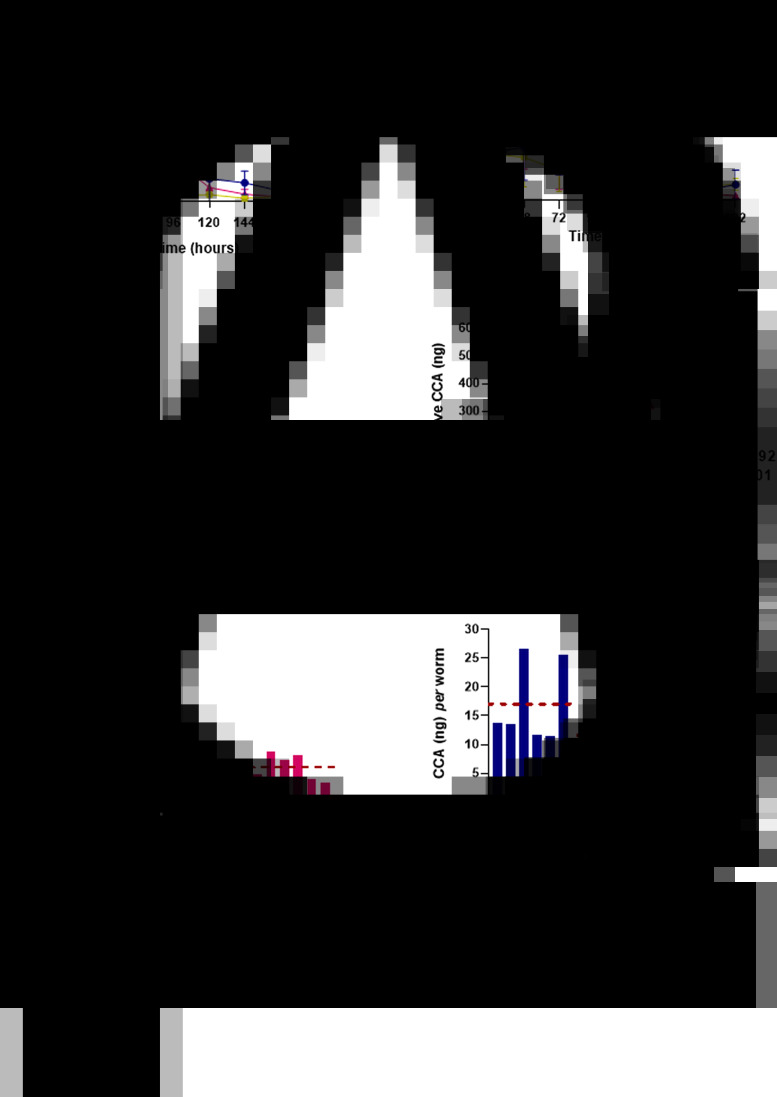
Mean concentration (ng/mL) of CAA (A) and CCA (B) *per* timepoint expressed *per* worm. Association of cumulative amount (ng) of CAA (C) and CCA (D) measured in culture medium over a period of 8 days *vs* total number of worms cultured. Cumulative amount (ng) of CAA (E) and CCA (F) produced over 8 days from *ex vivo* worm cultures expressed *per* worm. Dotted red line: mean circulating antigen per infection group. M: blue, circle; F: yellow, square; M + F: pink, triangle. Note the Y-axis have a different scale.

*Ex vivo* CAA levels were measured every 24 h and corrected *per* cultured worm (Fig. 5A). At 24 h, the M group excreted the highest CAA levels (34.7 ng/mL/worm s.d.: 38), which was 2.5 fold higher than M + F (13.8 ng/mL/worm s.d.: 16.9) and 2.3 fold higher than the F group (15.06 s.d.: 9.1). Interestingly, M and F groups showed a steady decline in CAA production, whereas the M + F group showed a peak at 72 h which was significantly higher than the F group (*P* = 0.01), but not higher than the M group. At the end of the *ex vivo* culture (day 8) CAA production had significantly declined but was still detectable for M and M + F groups (Fig. 5A).

A significant correlation was seen between the cumulative amount of CAA (ng) produced over 8 days of *ex vivo* culturing and the total number of worms cultured (*r* = 0.96; *P* < 0.001) where generally M and M + F groups had the highest cumulative excretion compared to the F group (*P* = 0.02) (Fig. 5C). Expressing the cumulative CAA production over 8 days divided by the number of worms *per* well, we observed the same pattern where M showed the highest mean cumulative CAA (123 ng/worm s.d.: 119), followed by the M + F group (79 ng/worm s.d.: 30) and the F group (30 ng/worm s.d.: 13) despite variability within the M group (Fig. 5E).

CCA concentrations detected by UCP-LF assay in culture media and measured every 24 h showed similar patterns as CAA per cultured worm, although overall concentrations were substantially lower (Fig. 5B). Generally, CCA concentrations *per* worm for the M group were slightly higher, and like for CAA, a peak of CCA was found at 72 h for the M + F group which was significantly higher than for the F group (*P* = 0.01). Similarly, a significant correlation was seen between the cumulative CCA produced and the number of worms cultured (*r* = 0.92; *P* < 0.001) where the M and M + F groups had the highest total CCA compared to the F group (*P* = 0.01; *P* = 0.03) (Fig. 5D). Expressing the cumulative CCA production over 8 days divided by the number of worms *per* well, the highest amounts of CCA were found in the M + F infection group (19 ng/worm s.d.: 9), followed by the M group (17 ng/worm s.d.: 7), and the F group (12 ng/worm s.d.: 3) (Fig. 5F).

## Discussion

In this study, we evaluated the excretion patterns of schistosome adult worm-related antigens *in vivo* (serum and urine CAA) and in *ex vivo* worm cultures (CAA and CCA) in male-only (M), female-only (F) and mixed male and female (M + F) schistosome infections. *In vivo*, CAA serum levels peaked at 6 weeks after infection, after which they declined. *Ex vivo* production of both CAA and CCA declined over time. Significant differences were observed both *in vivo* and *ex vivo* between sex of the parasite and antigen concentrations, with male worms excreting substantially higher levels than females during a single-sex infection.

It has been indicated that a lack of pairing may lead to developmental delay (Kunz, 2001; Pearce and Huang, 2015), potentially affecting the tegumental uptake characteristics and consequently their digestive activity and antigen regurgitation capacity (LoVerde *et al*., 2004). Interestingly, if females are separated from males after living in a paired condition, they have been reported to secrete more CAA and CCA than male worms (van Dam *et al*., 1996). This contrasts our findings, whereby we see that females secrete very little antigen (both CAA and CCA), lower than both M + F or males alone, although there was considerable variability. As such, we hypothesize that pairing potentially increases the metabolic demand of the egg-producing female worm, which has been described as being more metabolically active and capable of increasing erythrocyte ingestion up to 8.5 times more than males (Lawrence, 1973), leading to its darker colour and higher antigen regurgitation (van Dam *et al*., 1996). As antigen levels by single male worm infections do not significantly differ from paired mixed infections, the male worms do not appear to be limited in development by the absence of pairing.

Recovery rates of M and M + F infections corresponded well with other studies (19–39%, (Eloi-Santos *et al*., 1992; Smithers and Terry, 1965)). Much lower numbers of worms were recovered after female-only infection (F group), indicative of the higher vulnerability of the female worms as compared to the male worm or the female worm in the presence of the male. In M + F infections we found high levels of *Schistosoma* DNA in stool, but no significant correlation between DNA load and the number of eggs in the stool, the number of worms recovered, or antigen levels in serum or urine. Similarly, no significant correlation was observed between the total number of worms and the eggs per gram tissue or faeces. The limited range in worm number, the possible absence of *in vivo* mating pairs and the uneven male-female ratio may explain the lack of correlation. Although factors like worm fitness, maturity, pairs *vs* singles, host variation or time after infection may each affect either antigen production or antigen clearance, our results suggest that antigen detection is a more reliable reflection of burden of infection as opposed to egg excretion, particularly when the worm loads are low and chances of non-paired worms higher.

*In vivo* CAA excretion was detectable in both single-sex as well as M + F mixed infection, generally showing higher levels in serum than in urine, as previously described (van Dam *et al*., 1996). Moreover, levels of CAA increased until week 6 at which time parasites have reached sexual maturity (Agnew *et al*., 1995). After week 6 and particularly at week 14, serum levels slowly decline whereas urine levels dropped quicker. This seems to be in agreement with previous data (Barsoum *et al*., 1990; Agnew *et al*., 1995; Alan Wilson *et al*., 2006) that (serum) CAA may be a good correlate of worm burden. Variability in excretion of CAA *via* the kidneys, may be the cause of the observed dynamics in urine CAA and might explain the lack of correlation with worm burden reported in some studies (Deelder *et al*., 1980; van Dam *et al*., 1996). The multifactorial dynamics of serum and urine antigen concentration is also reflected by the large variation between individual mice, despite standardized conditions. The observation that antigen excretion varies between male and female worms in single-sex infections may potentially complicate the use of antigen levels to estimate numbers of worms particularly in low burden (natural) infections where single-sex infections may occur by chance.

*Ex vivo*, it was found a good correlation between CAA and CCA cumulative excretion levels and the number of worms. Antigen excretion was generally the highest during the first 24 hours and declined over time. The current data suggest that *ex vivo* cultured worms excrete more CAA than CCA, contrasting previous findings (van Dam *et al*., 1996). Worth mentioning is that at the study of Van Dam worms were used 7 weeks after (not monosexual) infection and they were cultured in a different medium, including feeding with erythrocytes. The different kinetics of CAA and CCA excretion that we found suggest a change in the antigenic composition of the vomitus, although these results should be interpreted with caution given the variability in antigen excretion between worms. Previously it has been reported that the loss of worm integrity leads to a strong release of antigen (Koslowski *et al*., 2017), but we could not reproduce this finding. However, we did note considerable interindividual differences, between mice and worms, in the level of antigen excretion. Rather, we only found a peak excretion of CCA in the mixed-infected group at 96 hours instead of higher excretions accompanied by high mortality (data not shown). A possible explanation for the observed peak could be the dying of relatively more worms, causing increased release of the gut content. Possibly, the use of a medium which better support the *ex vivo* survival of the worm, such as the addition of ascorbic acid, red blood cells or cholesterol, could improve the viability and thus the stability of antigen excretion over time (Wang *et al*., 2019).

Interestingly, we found spontaneous (non-viable) egg deposition in one single-sex female infected mouse, but no inflammation or granulomas in the liver and intestines were observed, which was in stark contrast with the mixed infections where egg deposition happens naturally (Fallon, 2000). Indeed, unisexual female schistosomes have been described to occasionally develop some differentiated vitellinocytes which produce normally or malformed shaped, but non-viable eggs in some mouse strains, also called incomplete parthenogenesis (Sahba and Malek, 1977; Shaw and Erasmus, 1981; Shaw, 1987).

Overall, we mapped the antigen excretion patterns of both male, female and mixed schistosomes in infected mice and found that male worms generally secrete higher amounts of CAA and CCA. The significant correlation between the number of worms and serum CAA levels supports previous data suggesting that this is a reliable diagnostic measure which can be used to estimate infection worm burden. Moreover, the consistent detection of CAA in serum also in female worm infections show promise for the development of a female-only controlled human infection model for schistosomiasis to complement the existing male-only model.

## Supplementary material

For supplementary material accompanying this paper visit https://doi.org/10.1017/S0031182021001839. click here to view supplementary material
