# Supplementary material for: Excretion patterns of Schistosoma mansoni antigens CCA and CAA by adult male and female worms, using a mouse model and ex vivo parasite cultures
Source: Parasitology. 2021 Nov 5;149(3):306–13. doi: 10.1017/S0031182021001839 (PMC10097511; doi:10.1017/S0031182021001839)

**Supplementary data**

| **Infection** | **Mouse** | | **Worm number** | | **Eggs liver** | **Eggs intestines** | **Eggs stool** | **DNA stool** |
| --- | --- | --- | --- | --- | --- | --- | --- | --- |
|  | Weight (gr) | Liver (gr) | Males | Females | *per* gram | *per*  gram | EPG | Ct |
| **Male_1** | 23.96 | 1.39 | 7 | 0 | 0 | 0 | 0 | 0 |
| **Male_2** | 23.7 | 1.18 | 4 | 0 | 0 | 0 | 0 | 0 |
| **Male_3** | 23.18 | 1.14 | 18 | 0 | 0 | 0 | 0 | 0 |
| **Male_4** | 20.83 | 1.14 | 11 | 0 | 0 | 0 | 0 | 0 |
| **Male_5** | 25.05 | 1.31 | 10 | 0 | 0 | 0 | 0 | 0 |
| **Male_6** | 24.36 | 1.36 | 1 | 0 | 0 | 0 | 0 | 0 |
| **Male_7** | 23.82 | N.A | N.A | N.A | N.A | N.A | N.A | 0 |
| **Male_8** | 23.02 | N.A | N.A | N.A | N.A | N.A | N.A | 0 |
| **Female_9** | 23.11 | 1.23 | 0 | 2 | 0 | 0 | 0 | 0 |
| **Female_10** | 23.03 | 0.92 | 0 | 3 | 0 | 0 | 0 | 0 |
| **Female_11** | 25.48 | 1.30 | 0 | 5 | 0 | 0 | 0 | 0 |
| **Female_12** | 25.07 | 1.49 | 0 | 2 | 0 | 0 | 0 | 0 |
| **Female_13** | 25.87 | 1.37 | 0 | 4 | 0 | 0 | 0 | 0 |
| **Female_14** | 24.46 | 1.32 | 0 | 2 | 2 | 0 | 0 | 0 |
| **Female_15** | 22.10 | N.A | N.A | N.A | N.A | N.A | N.A | 0 |
| **Female_16** | 23.51 | N.A | N.A | N.A | N.A | N.A | N.A | 0 |
| **Mixed_17** | 23.86 | 2.05 | 4 | 6 | 10,132 | 2,318 | 0 | 23.47 |
| **Mixed_18** | 22.00 | 1.98 | 6 | 6 | 11,886 | 2,506 | 40 | 20.94 |
| **Mixed_19** | 22.58 | 2.20 | 2 | 3 | 11,306 | 3,234 | 0 | 21.5 |
| **Mixed_20** | 22.54 | 2.50 | 5 | 8 | 7,540 | 2,495 | 0 | 21.07 |
| **Mixed_21** | 19.88 | 1.48 | 3 | 4 | 5,665 | 1,004 | 40 | 23.08 |
| **Mixed_22** | 24.25 | 1.87 | 3 | 3 | 10,161 | 2,690 | 0 | 22.04 |
| **Mixed_23** | 23.13 | N.A | N.A | N.A | N.A | N.A | N.A | 20.31 |
| **Mixed_24** | 19.43 | N.A | N.A | N.A | N.A | N.A | N.A | 20.39 |
| **C25** | 24.82 | 1.06 | 0 | 0 | 0 | 0 | 0 | 0 |
| **C26** | 21.99 | 0.99 | 0 | 0 | 0 | 0 | 0 | 0 |
| **C27** | 22.38 | 1.17 | 0 | 0 | 0 | 0 | 0 | 0 |
| **C28** | 21.47 | 1.09 | 0 | 0 | 0 | 0 | 0 | 0 |
| **C29** | 21.68 | N.A | N.A | N.A | N.A | N.A | N.A | 0 |
| **C30** | 21.83 | N.A | N.A | N.A | N.A | N.A | N.A | 0 |

**Table 1**. Overall data at perfusion point. Male (single infection with males); Female (single infection with females); Mixed (infection with males and females); C (controls, not infected). Eggs liver and intestines calculated *per* gram of tissue digested in KOH. Eggs in stool by Kato-Katz technique and DNA in stool by PCR. N.A data not available (see materials and methods).

**Figure 1**. CAA excretion patterns by UCP-LF assay in serum from each individual infected mice expressed as ng/ml.
A: Single infection with male cercariae, B: Single infection with female cercariae and C: Infection with male and female cercariae. Note the Y axis have different scales.


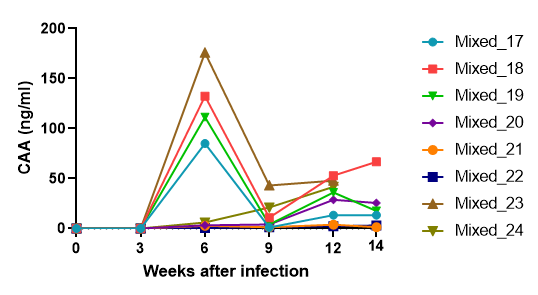

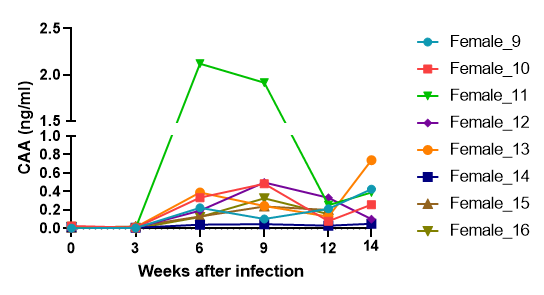

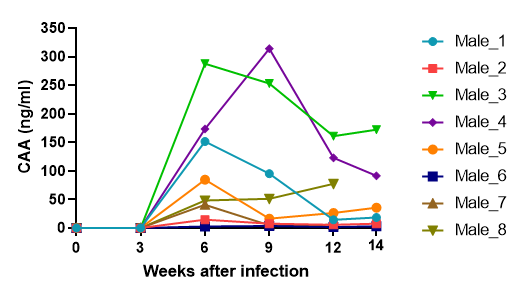

Supplement: Supplementary file 1 [file S0031182021001839sup001.docx]
